# Linking healthcare associated norovirus outbreaks: a molecular epidemiologic method for investigating transmission

**DOI:** 10.1186/1471-2334-6-108

**Published:** 2006-07-11

**Authors:** Ben A Lopman, Chris Gallimore, Jim J Gray, Ian B Vipond, Nick Andrews, Joyshri Sarangi, Mark H Reacher, David W Brown

**Affiliations:** 1 Department of Infectious Disease Epidemiology, Imperial College London, London, UK; 2 Centre for Infections, Health Protection Agency, London, UK; 3 Health Protection Agency, Bristol, UK; 4 Avon Health Protection Unit, Bristol, UK; 5 Health Protection Agency, Cambridge, UK

## Abstract

**Background:**

Noroviruses are highly infectious pathogens that cause gastroenteritis in the community and in semi-closed institutions such as hospitals. During outbreaks, multiple units within a hospital are often affected, and a major question for control programs is: are the affected units part of the same outbreak or are they unrelated transmission events? In practice, investigators often assume a transmission link based on epidemiological observations, rather than a systematic approach to tracing transmission.

Here, we present a combined molecular and statistical method for assessing:

1) whether observed clusters provide evidence of local transmission and

2) the probability that anecdotally|linked outbreaks truly shared a transmission event.

**Methods:**

76 healthcare associated outbreaks were observed in an active and prospective surveillance scheme of 15 hospitals in the county of Avon, England from April 2002 to March 2003. Viral RNA from 64 out of 76 specimens from distinct outbreaks was amplified by reverse transcription-PCR and was sequenced in the polymerase (ORF 1) and capsid (ORF 2) regions. The genetic diversity, at the nucleotide level, was analysed in relation to the epidemiological patterns.

**Results:**

Two out of four genetic and epidemiological clusters of outbreaks were unlikely to have occurred by chance alone, thus suggesting local transmission. There was anecdotal epidemiological evidence of a transmission link among 5 outbreaks pairs. By combining this epidemiological observation with viral sequence data, the evidence of a link remained convincing in 3 of these pairs. These results are sensitive to prior beliefs of the strength of epidemiological evidence especially when the outbreak strains are common in the background population.

**Conclusion:**

The evidence suggests that transmission between hospitals units does occur. Using the proposed criteria, certain hypothesized transmission links between outbreaks were supported while others were refuted. The combined molecular/epidemiologic approach presented here could be applied to other viral populations and potentially to other pathogens for a more thorough view of transmission.

## Background

Noroviruses are highly infectious organisms that cause an acute and short-lived gastroenteritis [1]. They are the most common etiologic agent associated with infectious intestinal disease [2,3]. Akin to many other gastroenteric pathogens, they are transmitted by the faecal-oral route. But, the virus also causes a high frequency of vomiting. During such an episode, virus is aerosolised. It can then be transmitted directly through the air or can settle and contaminate the surrounding environment or foodstuffs, later to be inadvertently swallowed [4]. Thus the transmission pathways of norovirus are complex and the application of molecular typing of the virus holds promise in furthering understanding of transmission [5]/

Molecular data have been used to describe the genetic diversity of norovirus in various national and regional populations [6-14]. These studies have collectively demonstrated these viruses are genetically diverse and dynamic, with new variants regularly replacing predominant strains. Moreover, molecular techniques are increasingly applied to assess suspected transmission links between outbreaks [5] A number of investigations illustrate this. Identical virus has been detected in patients as well as on an implicated food from a delicatessen meal [15]. A multi-state outbreak has been linked to a common source through tracing of common sequences in patients and a widely distributed oyster product [16]. Internationally-distributed raspberries have been linked through identical sequences following the distribution of a frozen fruit product throughout Europe and Canada [17]. These are only a few of the increasing number of reports which demonstrate the value of molecular genotyping. However, this approach has exclusively been used in the investigation of food and waterborne incidents which are a minority of norovirus outbreaks, at least in European countries [18].

Although these potentially linked outbreaks provide interesting anecdotes, from a statistical/sampling sense they may be dubious. The statistical question – what are the chances of detecting two outbreaks of the same genotype? – is purely data-driven, rather than hypothesis-driven.

Another problem with this approach, which is a general phenomenon in tracking pathogens, is that links will more often be hypothesised amongst rare types than common ones. The example of *Salmonella *highlights this. International outbreaks of Salmonella serotypes including Newport, [19] Anatum, [20] Saphra, [21] Bovismorbificans [22] and Agona [23], which represent relatively rare types, have been reported. In contrast, the linking of outbreaks caused by the major epidemic type, Salmonella enteritidis phage type 4, has proven extremely difficult [24].

In this paper, we will consider the question of using molecular data to assess norovirus transmission events in healthcare settings. The aim is to develop and test a system that is meaningful at the virus population-level, rather than to highlight rare events. The statistical methods that are applied here attempt to assess the significance of the molecular patterns in light of various epidemiological factors. Sound probabilistic criteria for linking of outbreaks and for assessing differences in molecular epidemiologic patterns with reference to place and time are presented.

Ethical approval for this study was obtained from the South West Multi-centre Research Ethics Committee. In nursing homes, executives provided written consent for the study to take place in their institutions; senior infection control nurses as well as microbiologists provided written consent for their hospital's participation.

## Methods

### The surveillance scheme and selection of strains for characterisation

Starting in April 2002, we conducted an active and systematic prospective study of gastroenteritis outbreaks in 171 inpatient units in 15 hospitals in the county of Avon, England [25]. Standard clinical definitions (of a case and a series of cases comprising an outbreak of gastroenteritis), uniform outbreak investigation, state-of-the-art diagnostics and null reporting ensured complete ascertainment of high quality epidemiological data. One or more faecal specimens were taken from affected persons in 122 of the 227 (53%) outbreaks. Of these, one or more specimen was positive for norovirus by RT-PCR [26] and/or ELISA [27] in 76 (63%) outbreaks. A single specimen from each norovirus-positive outbreak was selected for characterisation (except for the similarity criteria experiments described below).

### Typing scheme

Two regions of the norovirus genome were amplified and sequenced for each specimen. These regions were segments of the polymerase gene (open reading frame (ORF) 1) and the capsid gene (ORF 2). Ni/E3 primers were initially used to amplify the polymerase gene [26] and Mon 381/383 primers were used to amplify the capsid gene [28]. PCR products of Mon 381/383, at approximately 280 bases, were directly sequenced. Ni/E3 products are shorter at approximately 80 bases, and therefore required cloning and screening prior to sequencing. Initially, a third region (the inter-ORF1/ORF2 region) was also amplified and sequenced. However, initial studies demonstrated that the information added from sequencing this region did not affect conclusions and was therefore discontinued.

Genogroup II4 viruses were assigned a number-letter code based on their polymerase sequence and capsid sequence, respectively.

### Statistical testing

What is the probability that the observed patterns could be observed by chance alone?

The pathway of introduction of virus onto a unit can be broadly grouped into two categories: external (via introduction from the community by staff, patient, visitor or food) or internal (via transmission from another affected unit in the hospital). One method of testing the importance of internal transmission is to compare the viruses associated with an apparent cluster of outbreaks in a hospital to the whole population of viruses circulating in a reference population. It was hypothesised that if inter-hospital transmission played a significant role, the viral sequences from within a cluster of outbreaks would be more similar to each other than the viruses in reference population. Statistical testing was performed when space-time clusters of outbreaks met two criteria:

A. A cluster of outbreaks was defined as two or more unit outbreaks, occurring in the same hospital. Outbreaks were considered to be of the same cluster if the first date of onset in outbreak_n+1 _was before the last date of onset in outbreak_n_. Thus, a chain of outbreaks were considered to be part of the same cluster.

B. Within the cluster of outbreaks, there were two or more identical sequences from different outbreaks.

If the conditions in A and B were met, the probability that the sequences in the cluster could be randomly drawn from all virus sequences from the study population was tested. Viruses were grouped as belonging to the common sequence or other non-identical sequences. Fisher's exact test was used to compare the virus in the cluster to all variants identified in the study. If the test was significant (p < 0.05), it was concluded that there was evidence that viruses were being transmitted within the hospital.

What is the probability that outbreaks with anecdotal links had a transmission link based on viral sequence data?

In the present study, there were a 5 pairs of outbreaks with a known epidemiological link to another outbreak. For these linked outbreaks, the probability that the pair of viruses were truly linked was estimated, taking into consideration the background virus population. This probability was estimated using the following formula, the work-up of this formula is shown in Additional File #1.

P(x|Type = M) = ca_n_/(ca_n_+(1-c)b)

Where P(x|Type = M) is the probability that the second outbreak (of virus type 'M') came from the first outbreak.*b *is the proportion of viruses of type 'M' in the whole population.*a *is the probability that two viruses will differ by *n *or more nucleotides given that they are from the same outbreak. (The calculation of the similarity criteria (a) is described in the next section.) And *c *is prior estimate of the probability that the second outbreak has a transmission link to the first. The sensitivity of the probability estimates to the selection of the prior *c *will be presented. In the reported estimates, we assumed that c = 0.5, implying that it is equally likely that 1) the second outbreak came from the population and 2) the second outbreak came from the first case. When c = 0.5, the probability that outbreaks are linked simplifies to:

P(x|Type = M) = a_n_/(a_n_+b)

### Development of similarity criteria (a)

Data from the present study were combined with previous unpublished work performed by the Enteric Virus Unit to develop similarity criteria. Data were available from thirty-three other outbreak studies where multiple specimens were sequenced. Multiple viruses were sequenced from three outbreaks from the present study. These data were used to estimate the number of point mutations that would be expected from virus from the same outbreak. In the molecular analysis from the present study, 357 bases were sequenced. Therefore, the expected number of point mutations per 357 bases between two viruses that truly had a transmission link was calculated as follows:

*a *= 357 (M/[Σ L (s)])

where M = number of point mutations, L = sequence length (bases) and s = number of specimens sequenced.

## Results

### Virus population

Seventy-six outbreaks were selected for sequencing based on positive diagnostic results. RT-PCR amplification failed on 12 of these. Thus, virus from 64 separate outbreaks was characterised by genetic sequencing. Based on sequence from the polymerase and capsid, 61 of these viruses (95%) closely clustered with genogroup II4 (≥90% similarity with prototype Lorsdale strain). There were single detections of a genogroup I2, II3 II6. Fifty-eight of the 61 genogroup II4 viruses (95%) had the AATCTG motif that characterised the epidemic variant of 2002/03 [29]. Based on the polymerase region, there were 12 unique genogroup II4 sequences; based on the capsid region there were 16. In the polymerase, there were 2 predominant sequences associated with 30 outbreaks (sequence 1, 50% of total) and 14 (sequence 2, 23% of total) outbreaks. In the capsid, there were 45 identical sequences (sequence A, 74% of total); the rest of the Region C sequences were all unique (n = 16).

When the two regions were analysed together, there were a total of 26 unique sequences (Table 1). Henceforth variants are referred to by a number arbitrarily assigned to each unique *pol *sequence and a letter arbitrarily assigned to each unique *cap *sequence. The two most common variants were 1A associated with 23 outbreaks and variant 2B associated with 13 outbreaks (39% and 21 % of genogroup II4 outbreaks, respectively).

**Table 1:** Combined sequencing results of polymerase and capsid: genogroup II4 strains

		Capsid variant*																
		A	B	C	D	E	F	G	H	I	J	K	L	M	N	O	P	Total
Polymerase variant	1	23	1	1					1	1	1		1	1				30
	2	13														1		14
	3	1				1	1	1										4
	4	1										1					1	3
	5	2																2
	6				1													1
	7	1																1
	8	1																1
	9	1																1
	10	1																1
	11														1			1
	12	1																1

	Total	45	1	1	1	1	1	1	1	1	1	1	1	1	1	1	1	60

*One virus was not typed in Region A, which was variant Q in Region C (not shown)

**Figure 1:**
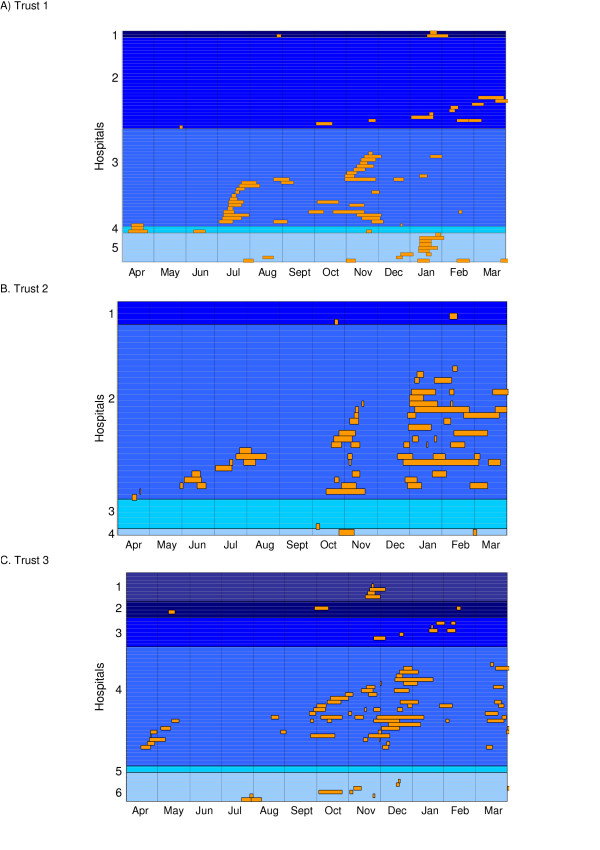
Gantt display of temporal patterns of gastroenteritis outbreaks. The varying shades of blue background represent hospitals within each NHS trust and each horizontal line represents an inpatient unit. Blue sections are 'outbreak-free periods' and orange sections are 'outbreak periods' (from the 1^st ^to the last date of onset). A high degree of temporal clustering can be observed in all Trusts. In other words, outbreaks do not often appear in isolation but rather many units are affected sequentially.

Hospital outbreaks appeared to cluster temporally, as shown in the Gantt charts in Figure 1. This figure includes all gastroenteritis outbreaks – including those where no specimens were available or were negative for noroviruses. Outbreaks exhibited a wintertime seasonality and also had a summertime peak – rather then being evenly distributed throughout the year (p = 0.001, Fisher's exact test).

### Similarity criteria

Combining data from the present study with previous work, sequences were available from multiple viruses from 36 outbreaks (Table 2). Amongst these, there were single nucleotide differences in a total of nine viruses relative to the consensus outbreak sequence. There were no outbreaks where sequences differed by more than one nucleotide.

**Table 2:** Studies that analysed within-outbreak sequence variation (not including mixed outbreaks caused by multiple genotypes), includes the present study (n = 3) and various other studies (n = 33) conducted by the Enteric Virus Unit, 2002–04*

Outbreak	Genotype	Primers^a^	Fragment length (bases)	Identical/sequenced^b^
1	GGII4	Ni/E3, Mon 381/383	357	4/4
2	GGII4	Ni/E3, Mon 381/383	357	4/4
3	GGII4	Ni/E3, Mon 381/383	357	4/5
4	GGI	SG1/D1	109	57/60
5	GGII^r^	Ni/E3	76	9/9
6	GGII4	Ni/E3	76	7/7
7	GGII1	Ni/E3	76	8/8
8	GGII4	Ni/E3	76	2/2
9	GGII4	Ni/E3	76	2/2
10	GI2	SG1/D1	109	2/2
11	GGII4	Ni/E3	76	2/2
12	GGII1	Ni/E3	76	4/4
13	GGII3	Ni/E3	76	3/3
14	GGI1	SG1/D1	109	2/3
15	GGII1	Ni/E3	76	4/4
16	GGI1	SG1/D1	109	2/2
17	GGII4	Ni/E3	76	2/2
18	GGI1	Ni/E3	76	2/2
19	GGII4	Ni/E3	76	2/2
20	GGII4	Ni/E3	76	3/3
21	GGII4	Ni/E3	76	2/2
22	GGII4	Ni/E3	76	4/4
23	GGII4	Ni/E3	76	2/2
24	GGI3	Ni/E3	76	2/2
25	GGII4	Ni/E3	76	2/2
26	GGII4	Ni/E3	76	2/2
27	GGII4	Ni/E3	76	1/2
28	GGII7	SG1/D1	109	1/2
29	GGI6	Ni/E3	76	2/2
30	GGII4	Ni/E3	76	2/2
31	GGI3	SG1/D1	109	2/2
32	GGII8	Ni/E3	76	2/2
33	GGII8	Ni/E3	76	4/4
34	GGII4	Ni/E3	76	1/2
35	GGI6	SG1/D1	109	1/2
36	GGII4	Ni/E3	76	2/2

Total			18678	157/166

^a^Ni/E3 and SG1/DI amplify the *pol *(ORF 1) region. Mon 381/383 amplify the *cap *(ORF2) region.

^b^'Non-identical' variants all differed by a single point mutation

^r ^Recombinant

*Short sequences within the RdRp can be used to differentiate between strains but genotyping relies on sequencing a region of the gene encoding the capsid. Also, sequencing regions of either the capsid or the RdRp will not identify recombinant strains. In this study, the characterisation of the genes encoding the RdRp and capsid was confirmed by sequencing a region spanning the ORF1/ORF2 junction, a common recombination site (data not shown).

These data were then used to set 'similarity criteria' (Table 3). Based on these data, if two viruses differed by a single nucleotide, there was a 17.2% chance they could be from the *same *outbreak. Reciprocally, there would be a 82.8% chance they were from *different *outbreaks. Summing the (diminishing) probability of 1, 2, 3, and 4 nucleotide changes (a+ a^2+ ^a^3^+ a^4^...) suggests that if viruses differed by one or more nucleotides there was a >80% chance that they were truly from separate outbreaks.

**Table 3:** Development of similarity criteria

Point mutations (n nucleotides)	Similarity probability formula	The probability (expressed as a percent) that two viruses will differ by *n *or more nucleotides given that they are from the same outbreak.	Average number of viral sequences that would have to be sequenced from the same outbreak to have 1 sequence with n nucleotide changes (1/(a)^n^)
1 nucleotide	a^n ^= a^1^	17.2%	6.2
2 nucleotides	a^n ^= a^2^	2.96%	38.5
3 nucleotides	a^n ^= a^3^	0.509%	Approx. 250
4 nucleotides	a^n ^= a^4^	0.088%	Approx. 1000

Any changes		< 20%	> 5

What is the probability that the observed molecular patterns could be observed by chance alone?

**Figure 2:**
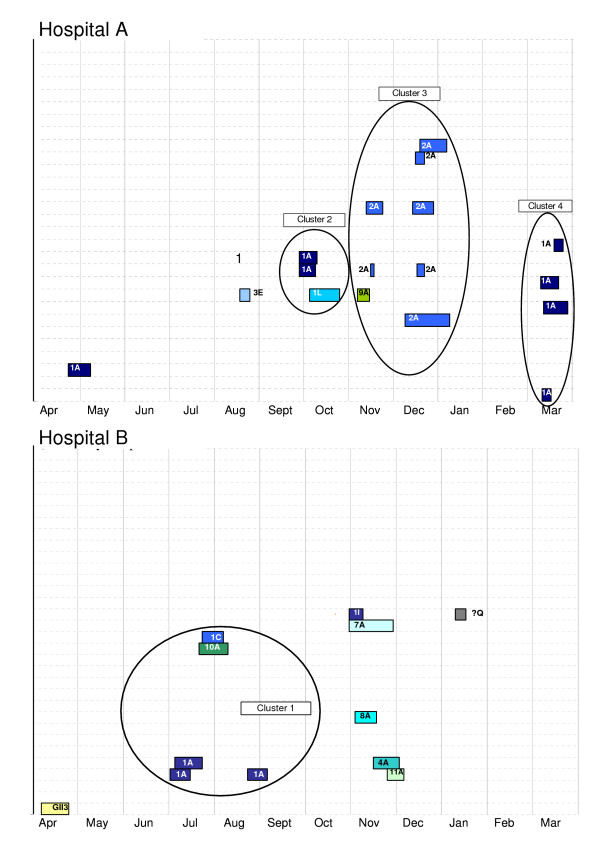
Characterised norovirus outbreaks in two hospitals in Avon England April 2002 to March 2003. Each row depicts the follow-up of a single hospital unit. Colored bars represent the period between the onset of illness in the first and last case in an outbreak where norovirus was characterised. Each unique norovirus sequence is represented by a different color. Series of outbreaks meeting the definition of a cluster are circled and were tested for statistical significance.

A total of four clusters were detected that met the definition proposed above (Figure 2). The first was in Hospital B (July/Aug). The other three occurred in the Hospital A in September-October, November-January and March. Although clusters 1 and 2 had higher proportions of 1A than in the population, the differences did not reach the level of statistical significance (perhaps due to the small numbers in the clusters)(Table 4). Clusters 3 and 4, however, did have significantly higher proportions of 2A and 1A (respectively) than would be expected by chance (Table 4).

**Table 4:** Probability that viruses in clusters of outbreaks differ from the population of circulating viruses (genogroup II4)

Cluster^a^	Common Sequence	Common sequence in cluster/total sequenced specimens in cluster	Common sequence in rest of population/total sequenced specimens in rest of population	Fisher's exact test (P-value)
1	1A	60% (3/5)	38% (20/55)	0.36
2	1A	67% (2/3)	38% (21/55)	0.33
3	2A	88% (7/8)	12% (6/52)	0.004
4	1A	100% (4/4)	34% (19/56)	0.018

^a^See Figure

What is the probability that outbreaks with anecdotal links have a common source?

There were a total of five pairs of outbreaks with anecdotal evidence of a transmission link between the events. These outbreaks and the links between them are described in Table 5. In three out of five of these outbreak-pairs the sequences in both *pol *region as well as *cap *region were identical. All of these pairs were genogroup II4, variant 1A – the most common variant detected in the outbreaks. Based on the probability formula, it was estimated that there was a 72% chance that these viruses shared a transmission link.

**Table 5:** Probability that anecdotally-linked outbreaks have a common source based on epidemiological and virological sequence data sequence.

Pair	Variant_x_	Variant_y_	Δ (bases)	Description of epidemiological link	a	b	Probability of transmission link
1	1A	1A	0	Doctor exposed on affected ward then worked on another ward while ill. Outbreak began on this ward 1 day later.	1.0	0.38 (23/60)	**72%**
2	3D	3E	3*	Transfer from nursing home into hospital (ward unspecified)	0.004	0.016 (1/60)	**20%**
3	1H	2M	4**	Transfer from hospital to nursing home of primary case	0.001	0.016 (1/60)	**6%**
4	1A	1A	0	Transfer from hospital to nursing home	1.0	0.38 (23/60)	**72%**
5	1A	1A	0	Transfer of patient from hospital affected to unaffected wards	1.0	0.38 (23/60)	**72%**

a Probability that the viruses could be drawn from the same outbreak basic on genetic similarity

b Probability that second virus would randomly be drawn from the viral population

c Probability that outbreaks with anecdotal links had a transmission link: P(x|Type = M) = ca/(ca+(1-c)b)

*3 nucleotide differences in the capsid

** 1 nucleotide difference in polymerase, 3 nucleotide differences in the capsid

**Figure 3:**
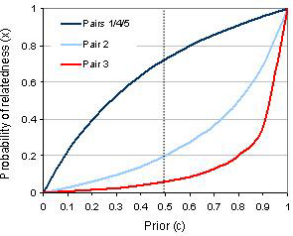
Sensitivity of the estimate of the probability of a transmission link between outbreaks given the range of prior assumptions of the link.

Another link, which involved transfer of a symptomatic patient from nursing home into hospital, was identical in the *pol *region but 3 bases different in the *cap *region. Finally, two outbreaks linked by the transfer of a patient from hospital to nursing home were different by a single base in the *pol *region and 3 bases in the *cap *region. Based on 3 and 4 nucleotide differences between viruses in these outbreak pairs, it was estimated that there was a less than 20% chance that these outbreaks truly had a transmission link. All the above probabilities are based on a prior estimate (c) of 0.5 which assumes an equal likelihood that the second outbreak came from the first and from the background population. Figure 3 illustrates the sensitivity of these probability estimates given different prior assumptions about the strength of the epidemiological link. When the genetic data is strongly suggestive of a link (such as pairs 1, 4 and 5), the probability estimate is very sensitive to the prior. These findings match the intuitive expectation that if, *a priori*, we believe a transmission link is unlikely on epidemiological grounds, a similar viral sequence should not be convincing. Conversely, if genetic data suggests that a link is unlikely (such as pairs 2 and 3) we must have an extremely high prior (c) based on epidemiological data, in order to conclude that true transmission link was likely to have occurred. Another interpretation is that when a virus sequence strain is common (as in Type 1A in pairs 1/4/5) epidemiological data must be strong in order to conclude that a transmission link is likely.

## Discussion

In this study, all the norovirus-associated outbreaks in a well-defined healthcare environment were sequenced. We have used this data to develop a system for assessing specific transmission links between outbreaks as well as the probability that the observed clusters were not due to chance.

95% of norovirus outbreaks were due to a single genogroup: II4 (phylogentically similar to what has been referred to by others as the Grimsby or Lordsdale clade). This is consistent with Gallimore et al's recognition that genogroup II4 variants are disproportionately associated with hospital outbreaks [30] and Koopmans' study that suggested that this same genotype is more frequently associated with outbreaks than sporadic community cases [13]. But a fundamental question remains unanswered: what are the unique biological characteristics of this genogroup that make it so predominant in healthcare settings?

It is clear, therefore, that the viruses causing these healthcare-associated outbreaks are not necessarily representative of all the circulating strains in the general community. However, we have no specific reason to believe that the strains that were amplified and sequenced were not representative of healthcare-associated norovirus outbreaks. Given the different genetic diversity in the community and healthcare facilities, we selected the healthcare-associated population of viruses as the reference for these analyses.

Historical data demonstrated that within an outbreak viruses were nearly identical; these data were then used to create similarity criteria. The subsequent analyses illustrate that combining virological and epidemiological evidence may give insight into transmission events. There was statistical evidence of greater similarity in clusters of outbreaks than would be expected from a random sample of the entire viral population. This suggests that transmission between hospital units is important.

A number of outbreak pairs that appeared to be linked based on anecdotal evidence proved to be caused by different viruses based on sequencing. Thus, *ad hoc *reports of links between outbreaks may not always be valid, especially when incidence is high. The opposite is also true: detection of identical viruses does not assure a direct link in transmission. Naturally, specific information on events can and will be taken into account. In this paper, we propose a method working towards systematising such information. We hope such methods will be used and taken forwards, perhaps in a Bayesian framework where one begins with a prior likelihood that events are linked, and the likelihood is updated based on virological or epidemiological findings. In this study we defined *a priori *what constituted epidemiological linkage, but, of course, many processes in infectious disease transmission are unobserved and therefore, possibility remains that, for example, that there were multiple introductions of the same virus.

In a hospital setting, where there is clearly potential for internal transmission, one may be drawn to the inference that identical virus necessarily implies a link. In fact, we estimate from this study that 15–20% of outbreaks where virus differs by a single base are really of the same outbreak. This limitation of inference based on genetic data applies our study as well as to other analysis. What this study adds is quantification of the level of inaccuracy.

The approach used here was based on extremely high resolution; inferences about transmission events were based on differences as small as a single nucleotide. However, this study is not the first to suggest that very small changes in the norovirus genome remain conserved geographically and temporally. From Maguire et al's analysis of the polymerase gene sequence in norovirus outbreaks in East Anglia, UK, it was clear that most outbreaks caused in a small geographical region were caused by a single variant and outbreaks in different locations formed different clusters [14].

These conclusions are meaningful because they are drawn from the context of a clearly defined population. Sections of the capsid and polymerase genes were sequenced – more than is often used to make inferences about the molecular epidemiology of noroviruses [5]. Furthermore, the sequencing of multiple viruses from within outbreaks provided a baseline to generate probabilistic statements of how likely it is that two viruses really are from a common source.

Despite these advantages, until a robust genotyping scheme is adopted for noroviruses (or whole genomes are analysed) there is always a distinct possibility that viruses that appear closely related are not really, and viruses that appear different by a few nucleotides in the targeted region are, in fact, related. The primers that have been developed for diagnostics target highly conserved regions [31,32] – less conserved regions may be more appropriate for these types of studies. Also, the rate of mutation is not precisely known for noroviruses, and the error rate in cloning/sequencing may have a strong bearing on results when small regions are sequenced. In this study, both the polymerase and capsid sequence were used to determine if outbreaks were linked whereas the underlying rates of mutation (i.e. uncertainly in the sequence data) were based almost solely on polymerase sequence data. Furthermore, two primer pairs were used to amplify different segments of the genome. The higher levels of variation in products of SG1/D1 primers may simply reflect greater variability in the target sequence as compared to the Ni/E3 primers.

One can only (or, perhaps, should only) make probabilistic statements about links between incidents. The literature is full of reports that assert links between norovirus [15-18] campylobacter [33-35] and salmonella [19-23] incidents. Clearly, an epidemiological link reinforced with characterisation data is highly suggestive. But what is the probability of randomly selected strains being the same? Background data on the diversity of circulating strains is needed to make this statistical assessment. As we have demonstrated here, once these data are available, they can be used to made clear probability statements about the likelihood that events are related.

## Conclusion

By systematic investigation of the virological and epidemiological characteristics of norovirus outbreaks in a hospital population, we were able to investigate transmission processes. The evidence suggests that transmission between hospitals units does occur. Using the proposed criteria, certain hypothesized transmission links between outbreaks were supported while others were refuted. The combined molecular/epidemiologic approach presented here could be applied to other viral populations and potentially to other pathogens for a more thorough view of transmission.

## Competing interests

The author(s) declare that they have no competing interests.

## Authors' contributions

BAL performed analyses, drafted manuscript and was involved in design of the study; CG performed genetic characterization experiments, JJG contributed to study design and genetic characterization experiments, IBV performed genetic characterization experiments and advised on study design, NA developed and advised on statistical methods, JS, MHR and DWB led the design of the study. All authors contributed to the drafting and revisions of the manuscript.

## Pre-publication history

The pre-publication history for this paper can be accessed here:



## Supplementary Material

Additional File 1 Appendix. Derivation of Statistical Methods: Probability of Transmission Links Click here for file
