# Supplementary material for: Linking healthcare associated norovirus outbreaks: a molecular epidemiologic method for investigating transmission
Source: BMC Infect Dis. 2006 Jul 11;6:108. doi: 10.1186/1471-2334-6-108 (PMC1539008; doi:10.1186/1471-2334-6-108)
Supplement: Additional File 1 — Appendix. Derivation of Statistical Methods: Probability of Transmission Links [file 1471-2334-6-108-S1.doc]

Appendix – Derivation of Statistical Methods: Probability of Transmission Links

Here we described the derivation of the formula of the probability that outbreaks with anecdotal links had a transmission link based on viral sequence data.

Suppose we get an initial outbreak followed by another outbreak which is linked epidemiologically (anecdotal link). Let x denote the event where the second outbreak came from the first and y the event when it did not.If we assume that the second case must either come from the first or from the background population then the probability the second case came from the background is P(y)=1-P(x). Before we know the type of the second case we have a prior probability for x (which is set as 0.5 for being equally likely the case came from the background or from the first outbreak). Let this prior probability be P(x)=c. We now observe the type of the second outbreak (type M) and wish to update P(x). From Bayes theorem:

P(x|Type=M) = P(x)P(Type=M|x) / P(Type=M) [1]

And

P(y|Type=M) = P(y)P(Type=M|y)/P(Type=M) [2]

Now we can calculate P(Type=M|x). This comes from the probabilities of differences in base pairs - which when both are Type A is effectively 1 (assuming that the viruses have not mutated in the observed chain of transmission). We then calculate P(Type=M|y) which comes from the proportion of type A in the population).If we let P(Type=M|x)=a and P(Type=M|y)=b. [4]

We know

P(x |Type=M) + P(y |Type=M) = 1. [5]

So we know

ca/P(Type=M) + (1-c)b/P(type=M) = 1 [6]

So we can calculate

P(Type=M) = ca + (1-c)b [7]

Subbing this back into the formula 6 above we get

P(x|Type=M) = ca/(ca+(1-c)b) [8]

and If c=0.5 we get

P(x |Type=M) = a/(a+b) [9]
